# The Artificial Pneumothorax Association and Journal

**Published:** 1914-04-18

**Authors:** Claude Lillingston

**Affiliations:** Gorleston-on-Sea


*To the Editor of* The Hospital.

### The Artificial Pneumothorax Association and Journal.

*To the Editor of* The Hospital.

Sir,—May I draw the attention of your readers to the existence of the International Association, "Pneumothorax Artificialis," which aims at the study and exchange of ideas on the subject of therapeutic pneumothorax? Professor Forlanini, honorary president of the Association, has kindly altered the plan of his journal, *Rivista delle Pubblicazioni sul Pneumotorace Terapeutico,* so as to meet the requirements of the Association's members.

Originally published in Italian only, it will in the future contain reviews of recent papers on pneumothorax in English, German, and French. It will be published at irregular intervals, and sent free to every member of the Association, the annual subscription to which is 8*s*. I shall be pleased to receive and forward this subscription to the Association's Secretary, Professor Carpi, and to supply any further information on the subject that it is in my power to give.—Yours faithfully,

Claude Lillingston, B.S.

Gorleston-on-Sea.

